# The virtue of innovation: innovation through the lenses of biological evolution

**DOI:** 10.1098/rsif.2014.1183

**Published:** 2015-02-06

**Authors:** Douglas B. Kell, Elena Lurie-Luke

**Affiliations:** 1 School of Chemistry and Manchester Institute of Biotechnology, The University of Manchester, Princess St., Manchester M1 7DN, UK; 2 Life Sciences Open Innovation, Procter and Gamble, Procter and Gamble Technical Centres Limited, Whitehall Lane, Egham TW20 9NW, UK

**Keywords:** innovation, evolutionary computing, philosophy of science

## Abstract

We rehearse the processes of innovation and discovery in general terms, using as our main metaphor the biological concept of an evolutionary fitness landscape. Incremental and disruptive innovations are seen, respectively, as successful searches carried out locally or more widely. They may also be understood as reflecting evolution by mutation (incremental) versus recombination (disruptive). We also bring a platonic view, focusing on virtue and memory. We use ‘virtue’ as a measure of efforts, including the knowledge required to come up with disruptive and incremental innovations, and ‘memory’ as a measure of their lifespan, i.e. how long they are remembered. Fostering innovation, in the evolutionary metaphor, means providing the wherewithal to promote novelty, good objective functions that one is trying to optimize, and means to improve one's knowledge of, and ability to navigate, the landscape one is searching. Recombination necessarily implies multi- or inter-disciplinarity. These principles are generic to all kinds of creativity, novel ideas formation and the development of new products and technologies.

Nothing is original… all creative work builds on what came before.

(Kleon, A: Steal like an artist. New York: Workman, 2012.)

The amount of eccentricity in a society has generally been proportional to the amount of genius, mental vigour, and moral courage which it contained. That so few now dare to be eccentric marks the chief danger of the time.

(John Stuart Mill, On Liberty, 1896.)

## 1. Introduction

The popularity of innovation as a buzzword can be traced back to the 1990s [1] and it has become one of the most overused terms, with a very clear trend to add it to any project, idea or description: companies mentioned some form of the word ‘innovation’ 33 528 times last year based on the analysis of their annual and quarterly reports, which was a 64% increase from 5 years previously (http://on.wsj.com/1aF6gpx), and (based on a search at www.amazon.com) more than 750 books with ‘innovation’ in the title have been published in the last three months.

Strictly speaking, innovation is not just finding a new way of doing something or discovering a new insight; it is about a translation of this insight into a specific application that would have either social or commercial impact: ‘an innovation is something original, new, and important in whatever field that breaks in to a market or society’ [2]. The study of innovation is an emerging interdisciplinary field with new journals, professional associations and university departments focusing on the study and breadth of the phenomenon [3]. For the purpose of this review, we wish to look at the essence of coming up with new and meaningful insights, be it an outcome of academic research and/or product development in industry. In addition to this, to illustrate the difference between disruption and incremental innovations, we have decided to approach this via conceptual basics and by going back to the ancient Greeks and in particular to Plato. This is because the fifth and fourth centuries BC in Athens were periods of extraordinary innovation in several fields: art, literature, architecture, political institutions, rhetoric, science and philosophy. As a result, Plato (like many other intellectuals of his time) attempted to explain the process of innovation (creativity) [4]. In doing so, he forged certain ideas that have had potency ever since. In this particular instance, we would like to reapply the term virtue as a description of a positive quality and in the context of a quality of generating new meaningful insights independently of where they are being generated—the virtue of innovation.

Given the sheer desirability (one might even say the ‘inevitability’ in Western culture) of innovation, whether in science, technology or any other field of human endeavour, we thus take it that the better we can understand those pathways of innovation, the more easily we can foster it and attain (acquire) the virtue of innovation.

Many metaphors for the process(es) of innovation are possible. However, in common with authors such as Schumpeter [5], Campbell [6], Nelson & Winter [7], Kauffman [8,9], the authors of a volume edited by Ziman [10] (and see [11]), Rivkin [12,13], Goldberg [14], Olsson & Frey [15], Frenken & Nuvolari [16,17], Khanafiah & Situngkir [18,19], Baldwin *et al*. [20], Hodgson [21,22], Arthur [23], Fleming & Szigety [24], Valverde *et al*. [25], Ganco & Hoetker [26], Caminati & Stabile [27], Geisendorf [28], Simonton [29,30], Johnson [31], Vermeij & Leigh [32], König *et al*. [33], Wagner [34], Solé *et al*. [35] and Gabora [36], we find that an evolutionary metaphor captures all of the necessary hallmarks of innovation in an accessible and accurate manner, and we set it out here. For example, both technologies and scientific discoveries follow evolutionary trends in the form of change, typically improvement, with time. A recent article in this journal [37] develops a similar theme, featuring nine separate hallmarks or commonalities of biological and other evolution, and in some ways, this article might be seen as a complement to it. The ‘now’ is caused by the ‘before’ and also influences what the ‘after’ will look like—these evolutionary stages are intimately connected. In systematic innovation, these progressions are referred to as trends of engineering system evolution (TESE). TESE postulates that all technological systems, from soap to aircraft engines, develop according to the same objective trends. In other words, the evolutionary paths for all different kinds of technologies are actually similar [38]. The ‘laws’ of technical systems evolution were discovered by G.S. Al'tshuller after reviewing thousands of USSR invention authorship certificates and foreign patent abstracts. Al'tshuller studied the way technical systems have been invented and modified over time and developed the theory of inventive problem-solving (TRIZ in Russian and TIPS in English) [39–42]. The TRIZ ideas sit very easily with the evolutionary metaphor, because TRIZ implies that most inventions are in fact ‘recombinations’ of existing principles, a theme that is a core focus of this article. (Biomimicry provides a similar and related example [43].)

**Figure 1.**
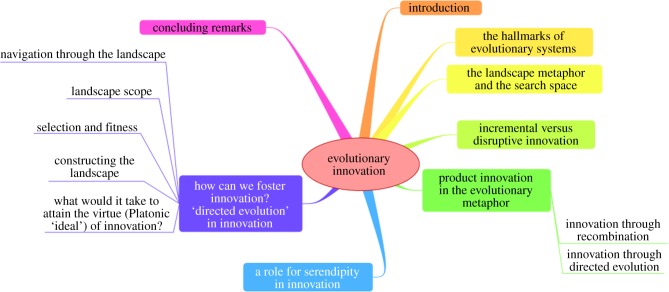
A ‘mind map’ [55] summarizing the contents of this review. (Online version in colour.)

We note that evolution comes in various forms, including natural (e.g. cosmological, biological) evolution, directed evolution (a term often used in improving molecules for biotechnology, see below), in computational modelling (e.g. mathematical algorithms known as evolutionary or genetic algorithms [44–46], including in improving software itself [47–49]), in cultural evolution [50] and even in the evolution of (scientific or other) ideas [51,52], in problem-solving [53] and in the diffusion of best clinical practice [54]. While we shall speak to many of these below, they all share some important, common features, and we begin with those. Figure 1 gives an overview of the manuscript.

## 2. The hallmarks of evolutionary systems

**Figure 2.**
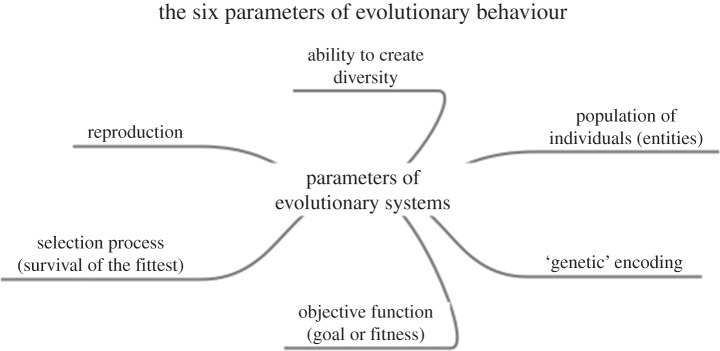
The chief attributes of any evolutionary system involving the creation of diversity, its fitness-based selection and the further production of diversity in subsequent generations.

For a system to exhibit the kinds of evolutionary behaviour we have in mind, which has been referred to as a ‘universal’ [56] or ‘generalized’ [57] Darwinism, the following six components must be present or pertain (figure 2).

— *A population of individuals* (*entities*), not all of whom are destined to survive (and none indefinitely), where each of whom inhabits some kind of bounded environment or ecosystem or universe (also known for some purposes as a ‘search space’).
— A ‘genetic’ *encoding* that is part of, and/or describes, the heritable properties of those individuals (entities). This will typically be in the form of a string of letters (such as nucleic acid bases in biological evolution), referred to as a chromosome, though other encodings, such as ‘tree-like’ encodings (discussed below) are also possible.
— An *objective function*, also known as a *goal* or a *fitness* that allows one to score the desirability or quality of the solution represented by the individual.
— A *selection scheme or step* that favours (but not exclusively) the survival of those that have ‘more’ of it or are closer to it. (There may sometimes be more than one objective function.)
— *The ability to perform reproduction* to create a new generation of individuals from those who survive long enough to reproduce.
— *Means of creating diversity as part of the reproductive process*, such that not every individual in a subsequent generation is identical to its parent or parents. Reproduction may be sexual (involving two or more parents) or asexual (involving only one). Typical means of creating diversity (in the genetic encoding) involve mutation (changing an element in an individual chromosome) or recombination (swapping parts of chromosomes between two or more parents). Reproduction (if sexual) may also be panmictic—any two individuals may mate—or more restricted (by ‘geographical’ or other means) such that a specific individual may mate only with a defined (more ‘local’) subpopulation.

Examples of how these six components may be represented in two different domains are given in table 1.

**Table 1.** Examples of hallmarks of evolutionary systems in biological and product development systems.

attributes	biological system	product/technology
the population of individuals	a population of individual organisms	candidate configurations within a platform product technology
a genetic encoding	the genome sequence	the main elements of the architecture of the technology, e.g. polymer structure
an objective function (a goal or a fitness)	survival	purposely driven innovation—a need to deliver a specific benefit, e.g. better cleaning
selection scheme	natural selection	market success
repurposing	moving to a new ecosystem/environment	reapplication of a technology in different areas, e.g. Nintendo—reapplication from the car industry to gaming
means of creating diversity as part of the reproductive process	mutation (changing an element in an individual chromosome) or recombination (swapping parts of chromosomes between two or more parents)	different product lines/next generation (e.g. iPhone 1, 2, 4, baby nappies → disposables, analogue → digital)

All of this is commonplace in the realm of natural Darwinian-style evolution. What is less recognized is that it maps generally onto the processes of engineering design and of evolution *sensu lato*. Here, it is useful to spend a little time on the concept of the ‘search space’ or universe that the individuals' ‘states’ may inhabit, in this case, the universe of all possible individuals. In the biological case, typically these are uncountably large, as they scale exponentially [52,58] with the number of *possibilities* of each ‘genetic’ element of the individual that can have different values or states. Thus, if the ‘universe’ is the list of all possible strings (i.e. sequences) of nucleic acids that are just 30 letters long (rather than say the human genome of approximately 3 000 000 000 bases or letters), and each position can have one of four letters (e.g. A, T, G and C), the ‘universe of possibilities’ is 4^30^ which is about 10^18^ [59], slightly greater than the lifetime in seconds of the known physical universe (*ca* 10^17^) [60]. Under these circumstances, we are unlikely to be able to assess every individual (sequence) that *could* exist.

## 3. The landscape metaphor and the search space

**Figure 3.**
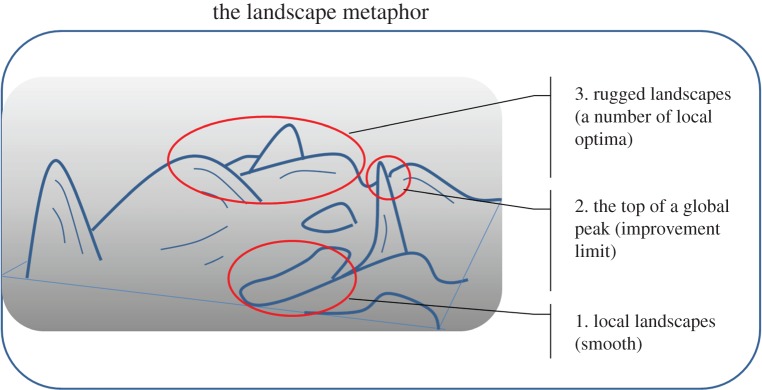
The fitness landscape metaphor for evolutionary search. In a Cartesian conception, ‘where’ one is in the (evolutionary) search space is encoded via the *XY*-coordinates, whereas fitness is encoded as the height or *Z*-coordinate. (Online version in colour.)

‘Landscapes’ as three-dimensional objects are easy to visualize in terms of two distinguishable properties (figure 3), namely where you are in *XY* space, used, for instance, to encode the genetic properties of an individual, and how high you are (in *Z* space, used to encode the fitness). Thus, it is assumed that the landscape reflects a metric in which things that are close to each other in *XY* space are close to each other in genetic sequence. In natural evolution, the landscape metaphor derives from Wright [61]. Although the landscape metaphor is not entirely perfect (e.g. how it looks or functions does depend upon what kinds of ‘moves’ or means of transport are available [62], and it does not easily reflect more than one fitness (there is only one ‘height’), we do find it extremely helpful in understanding the essential issues of evolutionary search and improvement, as well as being relevant for both academic and industry domains, and we shall retain it. Of course for non-biological domains, such as product innovation, see below, the non-fitness axes of the landscape reflect some more generalized ‘distance’, whether conceptual or technological.

**Figure 4.**
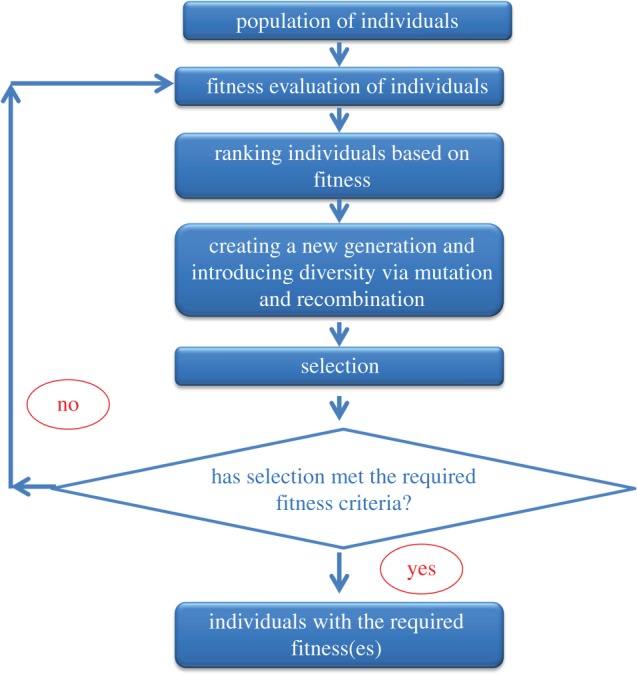
A flowchart describing a typical evolutionary algorithm. (Online version in colour.)

Thus, we propose to use the landscape to represent the ‘universe of possibilities’, and individuals, whether they be ideas, products, biological organisms or anything else (table 2), each occupy a particular (*XY*) position in that space, and that position has an associated fitness (height). If then the aim is to create (or breed) an individual with a greater fitness or height, and all one knows is the places that other individuals have occupied and what their fitnesses are [63], the ‘game’ is to search around the landscape for places with a greater height, and to do so in the shortest time (i.e. using the fewest individuals or the lowest number of attempts; figure 4). This also leads to *three* interlinked concepts. The *first* is that locally landscapes are reasonably smooth: if one goes in a certain direction and one goes up a slope (i.e. becomes fitter), a second move in the same Cartesian *XY*-direction is also likely to be (further) up. In terms of the product/technology innovation, this concept translates into a gradual improvement of a specific product property, for example development of better absorptive materials. An improvement of absorbent materials could be considered in a three-dimensional space in terms of thickness of material (*X*), flexibility (*Y*) and absorbent capacity (fitness, *Z*). Absorbency is a key function in diapers or nappies. There were a number of different shifts along the landscape that led to progress from the nineteenth century. The earliest from these times consisted of a cotton material, held in place with a fastening; this led to a two-part system of a disposable pad (cellulose wadding covered with cotton wool) and an outer plastic, adjustable garment with press-studs/snaps) developed by Valerie Hunter Gordon in 1947 (http://bit.ly/1zLHe19) and to today's technology based on superabsorbent polymers with multiple absorption zones [64].

**Table 2.** Landscape metaphor and search space.

*landscape metaphor*	biological system	product/technology
concepts defining a landscape		
1. Local landscapes	relationship between genotype and phenotype for closely related organisms, typically from the same species	improvement of a specific parameter, e.g. looking for a way to improve absorbent properties (absorbency landscape)
2. The limitations (boundaries) within the function changes (it cannot continue indefinitely)	limitations of a particular material being evolved (e.g. bone)	boundaries to property improvement, e.g. a reduction of weight of packaging will have a limit (no weightless materials)
3. Rugged landscapes	Sewall Wright's fitness landscape [61]	many products that are quite similar in performance may be based on very different technologies
navigation through the landscape		
4. Exploitation	local changes in genome and phenotype, typically by mutation	continuous (incremental) product improvements
5. Exploration	more substantial changes in genotype and phenotype, e.g. by recombination, horizontal gene transfer	seeking radically different products or product designs from other industries or application fields

The *second concept* is that this ‘local’ improvement cannot continue indefinitely, such that in time one reaches the top of a local peak (Snowdon, if you will; for non-UK readers, this is a hill of some 1085 m height), somewhat equivalent to a ‘dominant design’ in the product world [65]. The development (up to a limit) in a property's improvement can be illustrated by a rising trend in a reduction of weight of packaging materials to improve sustainability; however, there will be a limit to a weight reduction (there are no weightless materials). Another well-known example of this concept is a reduction of transistors' dimensions to improve their performance, and reduce power and cost per transistor—in the past three decades, the size of the transistor has moved from microscale to nanoscale—3 μm in 1980 to 22 nm in 2010 (http://intel.ly/1nMLOqM).

Clearly (staying with the ‘actual’ landscape, the biosphere of planet earth), there are higher peaks such as Mont Blanc (4810 m) in the Alps or Everest (8848 m) in the Himalaya, but one can only get to them by passing through regions of *lower* fitness (‘reculer pour mieux sauter’ [66]). Thus, the *third* key feature is that landscapes are ‘rugged’, involving many local optima, and that it may indeed be possible to characterize that ruggedness. Staying with the product packaging landscape, there could be a number of ways of how to improve this, for example using (i) lighter materials; (ii) better dispensing system; (iii) multi-functional packaging materials (e.g. packaging materials with antibacterial properties, smart packaging materials, etc.). The landscape ruggedness describes or reflects the trade-offs involved.

Finally, it is important to note how we search a landscape. Searching a landscape necessarily involves a blend between exploitation (‘going upwards’, in case you are on the slopes of Everest and not Snowdon) and exploration (looking further afield, possibly with a greater risk of failure but also perhaps with a greater chance of a notable success). Exploration and exploitation also involve a judicious blend between hypothesis-dependent and data-driven strategies [67]. This distinction between exploitation and exploration also arguably reflects *the distinction between incremental and disruptive innovation* (see later; table 3).

**Table 3.** Some examples of incremental and disruptive innovations in the context of the evolutionary metaphor.

type of innovations	example	nature of ‘mutation’ or ‘recombination’	function	landscape distances covered
disruptive	three-dimensional printing	bringing together printing, materials science and three-dimensional design	making a three-dimensional solid object	broad: from printing to tissue engineering
disruptive	synthetic textile dyes	moving away from natural products, bringing in entirely new (synthetic) compounds	more effective and novel colorations	broad: very different in terms of chemical space
disruptive	synthetic detergent	moving away from soap use in clothes washing, bringing in entirely new (synthetic) compounds	more effective cleaning aid—obsolete soap powders	broad: cross-disciplinary research and novel chemical space
disruptive	fabric odour elimination	creating a new product category—fabric odour elimination and freshness	providing a different benefit versus air-freshening products, i.e. odour elimination and freshness by actually capturing odours versus masking them with perfumes	broad: from a technology to marketing
disruptive	Velcro antibacterial fasteners	adding a new functionality	brand fasteners with antibacterial silver treatment to help reduce the risk of cross-contamination	broad: fasteners and anti-microbial
disruptive	smart packaging	adding a new functionality	smart packaging that can verify effective oxygen removal from food packages	broad: responsive materials, printed electronics, equipment design and systems integration
disruptive	laser keyboard	conversion of any surface into a keyboard	removing the need to have a physical keyboard (bridging between virtual reality and practical application)	broad: laser technology, design and systems integration
disruptive	Google Glass	wearable computer	Google Glass displays information in a smartphone-like hands-free format and communicates with the Internet via natural language voice commands	broad: material science, electronics, design and systems integration
disruptive	biopulping (paper making)	alternative process—using natural processes instead of chemicals	biopulping is based on enzymes that selectively degrade and dissolve the wood gluing compounds and provides a more environmentally friendly process	broad: biology, chemistry and engineering
incremental	new flavour of a sport drink	changing of flavour	expanding choice	local: sensorial/taste
incremental	new shape bottle	changing of shape	expanding choice	local: packaging materials
incremental	new textile print pattern	changing of colour and pattern	expanding choice	local: textiles design
incremental	aerodynamic wind- resistant umbrella	improving performance	improving the aerodynamic properties to withstand wind	local: design
incremental	balcony bridge planters	expanding usage occasions	pot design that enables it to be put on a balcony rail	local: design
incremental	self-stirring mug	no need for a spoon	the self-stirring mug has in-build stirring device.	local: design

## 4. Incremental versus disruptive innovation

In the previous sections, we looked at the process of innovation as an evolutionary process and also used the landscape metaphor to demonstrate differences between the processes leading to incremental and/or disruptive innovations. Taking into account these differences before beginning a project, it is very important to define what type of innovation one would like to pursue as it will help to develop a relevant research plan, select appropriate methodologies and also build the right expectations, both in terms of time and appraisal (recognition). In a way, it is similar to choosing a good problem to work on [68] as it can lead to generating new knowledge, technologies or/and actionable insights. Alon's paper [68] provides a very eloquent framework (the ‘feasibility-interest diagram’) to assist with problem selection, and also highlights the importance of discussions between teachers (supervisors) and students (researchers). Discussions, for example dialogues between teacher and student and/or between scholars, have been a part of the learning process (knowledge dissemination) since ancient times and throughout different cultures. One of best exemplifications of this is Plato's dialogues that were used in more than 30 of his works [69]. The dialogues as a form have been used to teach a range of disciplines, including philosophy, religion, ethics, logic, mathematics, etc. [70]. The above has prompted us to consider the development of a framework based on a philosophical metaphor that could help with choosing a type of innovation for the research project. This framework is designed using two axes (variables). The first variable deals with the essence of the innovation type and the second one is the function time.

To describe the essence of different types of innovation, we decided to use Plato's deliberations on virtues, in particular his work ‘*Meno*’, in which Socrates and Meno discussed what human virtue is in general, rather than particular virtues, such as justice or temperance and whether it can be taught [71]. Our choice of using ‘virtue’ to dimensionalize innovation in the philosophical context was driven by the following factors (argument). On the one hand, virtues are positive qualities and the foundation of good moral being; in a similar way, innovation, independently of being disruptive or incremental, is a positive trait indicating some success of the research programme. From another side, it provides an opportunity to differentiate between incremental and disruptive innovation via different types of knowledge that would be required to attain virtue. In *Meno*, Plato looks at virtue as a form of knowledge and then develops it into a more holistic concept where virtue encompasses knowledge and ‘something less—true belief’ [71]. True beliefs can be useful as knowledge, but they often fail to ‘stay in their place’ and must be ‘tethered’ by what he calls *aitias logismos* (the calculation of reason, or reasoned explanation) [72]. Thus, virtue can be viewed variously as true virtue and ordinary or civic virtue. True virtue is based on knowledge and gets it right all the time, i.e. true virtue knows why it is right, whereas ordinary virtue is based on habit. In regard to innovation, disruptive innovation can be compared with true virtue that requires both knowledge and time to acquire it, whereas incremental innovation can be associated with civic virtue that is based on conditioning and could be subject to change. Attaining true virtue could be a lengthy process: incremental innovation is often referred to as ‘continuous improvement’, with releases occurring in under a year, whereas it typically takes more than a decade to develop a breakthrough piece of research into a translational outcome [73] or to come up with a disruptive technology, for example it took more than a decade of research from the recognition that restriction enzymes could be used to make recombinant DNA [74], which arguably initiated the era of modern biotechnology, until the availability of recombinant insulin, the first main biotech product [75]. Similarly, it took Dyson more than 10 years (http://bit.ly/1tUDFFz) to redefine vacuum cleaner technology, following his recognition of the potential of cyclones.

To distinguish between disruptive and incremental innovations as a function of time, we have turned to another philosophical text by Plato, The Symposium. The Symposium is also written in the form of a dialogue, which takes place at a symposium or drinking party in the house of the tragedian Agathon at Athens where a group of men, including Socrates examined the subject of love in a sequence of different speeches [76]. In particular, we would like to focus on Socrates's speech where he retells his conversation with Diotima, a female philosopher and priestess [77]. In her speech, she links the desire of immortality with the nature and function of love. Immortality in the true sense can be achieved only by gods, and humans can have only ‘surrogate immortality’ by leaving something to be remembered after death—‘a continuation of our existence after death’ with memory is the primary vehicle to achieve immortality [4]. For example, physical immortality is remembrance through having children; remembrance for various achievements, for example inventors, poets, legislators, etc., and remembering for wisdom. While the first two categories deal with human memory, the third category, remembering for wisdom, includes not only humans, but also gods and, because gods are immortal, such memory is forever secure [4]. The group that achieves the latter immortality is philosophers that give birth to ‘true’ virtue. This memory construct can be applied to differentiate between two types of innovation. Disruptive innovation has a longer life cycle than incremental innovation and hence is associated with a longer memory and the potential to achieve immortality, for example, how long one would remember new packaging or a new flavour of soft drink (incremental innovation) versus breakthrough products, for example pill cameras ‘PillCams’ (http://bit.ly/UbSp2i) or synthetic detergent technology [78] (disruptive innovation).

There are of course hundreds of examples of the lengthy time between a rather abstract or fundamental scientific or mathematical discovery and its true exploitation. Thus, Dirac first brought e-spin into the logical structure of quantum mechanics, and after the best part of a century, his equation (once seen as a piece of mathematical research with no relevance to everyday life) has become the theoretical basis of the multi-billion dollar electronics industry [79], whereas the positron, simply postulated as part of the antimatter necessary for the theory, is now widely used (via positron-emission tomography) in non-invasive diagnostic imaging procedures [79].

There is also a clear distinction between incremental and disruptive innovation within the framework of the landscape metaphor. ‘Incremental’ innovation involves local improvements that do not stray far from the previous generation while improving their fitness slightly. By contrast, disruptive innovation typically involves a substantial change in both the ‘genotype (i.e. the nature of the individual/product) and its fitness’. In the book *‘The innovator's dilemma’* [80], Christensen describes two major types of innovations—(i) those based on ‘sustaining’ technology and (ii) those based on ‘disruptive’ technology. Sustaining technology innovations are characterized as providing superior performance, having strong customer or consumer focus and being based on either existing or new technology in the company. Disruptive technologies have new performance measures, different value elements and are often not ‘needed’ by current customers.

In science, the distinction between what he called ‘normal’ (incremental) and ‘revolutionary’ (disruptive) science is of course the cornerstone of Thomas Kuhn's famous analysis of ‘the structure of scientific revolutions' [81]. This said, the ‘revolutionary’ changes in the Kuhnian scheme do not necessarily occur ‘quickly’; they sometimes follow a timely evolutionary ‘landscape’ of their own. It is also possible to argue, or at least to recognize, that the iterative interplay between theory and experiment driving scientific advances also involves a certain kind of recombination.

## 5. Product innovation in the evolutionary metaphor

Applying these ideas to the design of a new product (table 3), it is easy to see the objective functions or fitnesses as these are set (and evaluated) by the experimenter (developer). Thus, the ‘fitness’ of a new product is likely to involve profits from increasing the share of an existing market or from penetrating novel markets, and may include subobjectives that lead to it, such as cost reduction of manufacture for undiminished quality. In a similar vein, as nicely set out by Goldberg [14], what a competent strategy requires follows precisely from this analysis. First, we need to decompose hard problems into easier ones (‘building blocks’), ensure by selection and artful decision-making both that we have a plentiful supply of quality (i.e. ‘solved’) versions of these, then recognize that the production of novelty overall relies heavily on the assembly of these preformed components in novel ways. We also note the literature of product development that recognizes that the need for incremental versus disruptive innovation is itself a function of the external operating conditions [82].

In a similar vein, and while the distinction is somewhat arbitrary, it is possible to argue that incremental innovation is more akin to mutation (a local change involving only one ‘parent’), whereas disruption requires the introduction of new material or ideas from afar, thus involving recombination (more than one parent and a ‘sexual’ element).

For example, three-dimensional printing started in the 1990s as a way of rapid prototyping development, where computer-generated three-dimensional models can be transformed into physical objects using a layer printing process [83]. It started with the ‘printing’ landscape with the fitness function being a production of three-dimensional objects. Advances in technology development in the areas of additive manufacturing machinery, material development and tissue engineering have enabled us to reduce the costs of three-dimensional printing and move it to the commodity domain as well to add versatility of different applications. This led to expanding the original ‘printing’ landscape to a number of different landscapes including ones where the fitness has become an ability to produce nanoscale-size objects [84] or tissue-like biological constructs [85]. Thus, the new landscape presents a combination of individual landscapes that are not ‘close’ in any real sense to what went before. This, however, is not the case for incremental innovation that typically operates within a single (and narrower part of the) landscape. For instance, sports drinks represent a fast-growing consumer category, and flavours play an important role in designing novel sports drinks. Staying hydrated during and after physical exercise is seen as an important factor for one's overall health [86], and thus the desire to ensure that this is done. This in turn leads to an increase in the variety of different flavours that are introduced. However, within the landscape metaphor, this variety of flavours is constrained to a single fitness function and generally the rest of the product is not changed. The search for novelty is local and incremental.

We here give some examples of the kinds of innovation that clearly come from optimizing and recombining elements from different disciplines or domains of knowledge, or even different domains of the same area.

### 5.1. Innovation through recombination

#### 5.1.1. Systematic DNA sequencing

As is well known, following the development of systematic DNA sequencing [87–89], there was an explosion in our ability to sequence nucleic acid bases that increased for many years at a rate similar to that of Moore's Law until another disruptive innovation, Solexa or Illumina sequencing [90], was invented. This brought together three essential, known, ideas (i) the ability to immobilize single-stranded DNA [91], (ii) the ability to incorporate fluorescent versions of nucleic acid bases during DNA sequencing-by-synthesis [92,93], and (iii) the ability to measure fluorescence from very small numbers of spatially separate molecules [94,95]. Rates of nucleic acid sequencing are tens of orders of magnitude greater (or costs per base equivalently lower) than those in 1977 [96–98], and they are likely to increase further as nanopore-based sequencing comes in [99,100].

#### 5.1.2. Synthetic fibre

Innovation can be facilitated by combining expertise from different domains (industries), for example, creating a new fibre by bringing together petroleum-based fibre expertise and biotechnology expertise. DuPont and Genencor collaborated to develop a biotechnological process in which bacteria were made to produce a required material. The gene-engineered bacteria converted glucose into propanediol which was used by DuPont to make the new fibre, Sorona, and create a more environmentally sustainable process by using starch instead of petrochemicals [101,102]. The same is true for bioisoprene [103].

### 5.2. Innovation through directed evolution

#### 5.2.1. Directed evolution of proteins

A particularly clear example of innovation as evolution comes from the field known as directed evolution. In biology, genes encode proteins according to a fixed mapping (the ‘genetic code’) whereby particular triplets of bases, known as codons, encode the sequence of a protein that is then made by suitable hosts cells. There are 4^3^ = 64 triplets and 20 natural amino acids, so in some cases, more than one triplet encodes a particular amino acid, whereas three of the 64 codons, known as stop codons, encode a signal to stop reading the DNA. The aim, as ever, is to move round the search space by creating variance in the sequence while measuring the fitness in terms of properties such as the ability to catalyse, as ever, greater rates of a particular (bio)chemical reaction. (Other objectives, whose values also vary with the sequence, might include the ability to resist ‘high’ temperature or to tolerate non-aqueous solvents.)

The innovation necessarily follows directly from the vastness of the search spaces. As rehearsed previously [52], using only the 20 ‘common’ amino acids, the number of sequence variants for *M* substitutions in a given protein of *N* amino acids is 19 *M*.*N*!/(*N* − *M*)!*M*! [104]. For a protein of 300 amino acids with changes in just one, two and three amino acids this is 5700, *ca* 16 million and *ca* 30 billion. Even for a very small protein of *N* = 50 amino acids, the number of variants (20^50^) is some 10^65^! The same combinatorial formula applies to finding the subset of *k* enzymes out of *n* that one might wish to change for some benefit; if *n* is 1200 (a reasonable, even slightly low, number for metabolism [105–107]), for *k* = 1, 2, 3, 4, 5 and 6, these numbers are 1200, 719 400, 2.87 × 10^8^, 8.6 × 10^10^, 2.06 × 10^13^ and 4.1 × 10^15^. Thus, there is very little chance that any individual protein has been made during natural evolution.

What distinguishes natural evolution from directed evolution is that the selection is not done on the basis of any biological survival ability of the host organism but simply on the value *to an external agency* (the human experimenter) seeking a better biocatalyst (or whatever the product may be). While this is of course equally true in the human breeding of organisms whether say for agricultural purposes [108] or as domestic animals, it is probably the ‘molecular breeding’ or molecular evolution of proteins and other macromolecules that provides the clearest example of how evolution links to innovation.

Thus, we have examples of the role of recombination [109–111], fitness landscapes [110,112–114] and landscape ruggedness [110,115–117], neutrality [118,119], epistasis [113,120–127], optimal mutation rates [128], the benefits of genotypic knowledge [129,130] and diminishing returns [131]. The ability to synthesize proteins in a principled manner via the methods of synthetic biology opens up many possibilities here [132–134]. This said, as well as recombination or horizontal gene transfer, a hallmark of natural evolution is the production of random mutations; whether they are useful (selected) or not may be seen as a form of serendipity.

#### 5.2.2. Directed evolution of a synthetic detergent

Although the concept of ‘directed evolution’ is usually applied to proteins, at least in biology, similar phenomena can be observed in the ‘evolution’ of novel substances or recipes of other kinds [37]. The invention of synthetic detergents was driven by the need for a cleaning agent that would address the drawbacks linked to soap use, i.e. (i) the formation of a residue (‘curds’) that was a result of the reaction of soap with minerals salts, and (ii) the need for an alternative raw material to fats—indeed, the shortage of fats during the First World War drove German scientists to develop the first synthetic detergents. These detergents were of the short-chain alkyl naphthalene sulfonate type which were originally used in the textile industry as wetting agents. P&G scientists recognized the opportunities for using these agents and in 1932 P&G negotiated a licence to develop and market alkyl sulfates as synthetic detergents in the household and laundry fields. This technology led to the launch of a new synthetic detergent (‘Dreft’) in 1933. It was an innovative but limited laundry detergent, in that it did clean clothes well in hard water but was not able to clean heavily soiled clothes. Over much of the 1930s scientists worked to address this issue and developed alkyl-sulfate-based detergents capable of heavy cleaning: they tried building the surfactant with numerous compounds, they mixed soaps with synthetic detergents, they heated, cooled, extruded, pulverized and flaked any number of formulas, none of which proved satisfactory. Despite repeated frustrations, David Byerly refused to shelve the research and persisted. His efforts were further hampered by the outbreak of World War II, which led to shortages of raw materials, and the need to convert some processes to military supply and to reformulate many products because of rationing. Despite this, by 1941, Byerly had concluded that the best ‘builder’ (chemical compounds that soften water by removing cations) was sodium tripolyphosphate and had a counterintuitive breakthrough. All previous research on soaps and detergents had shown that reducing the amount of builder in a formula yielded a less harsh product (and it was the harshness of products with builder that hamstrung the project for so many years). Like his predecessors and colleagues, Byerly at first tried to keep the proportion of surfactant—the actual cleaning agent—as high as possible. But when he inverted the ratio by boosting the level of builder well above the amount of surfactant, he got a surprising result: the detergent cleaned well without leaving clothes stiff and harsh. Byerly determined that the correct formula was one part active detergent, alkyl sulfate, to three parts builder, sodium tripolyphosphate. This was a turning point and by mid-1945, a new formula and process were developed; Tide was chosen as the name of the product that was introduced to a test market in the US in 1946 [135].

## 6. A role for serendipity in innovation

The word ‘serendipity’ was coined by Horace Walpole from the Persian fairy tale *The three princes of Serendip* (Serendip is an ancient name for what is now Sri Lanka), whose heroes ‘were always making discoveries, by accidents and sagacity, of things they were not in quest of’. In modern terms, it might be seen as obtaining a finding that was not part of the expected outcome of a hypothesis-dependent experiment [52,67,136]. There are many well-known examples in science [34,137], although the pathway of innovation can be tortuous. From an evolutionary perspective, serendipitous events may be seen as random and fortuitous mutations or recombinations. The example of penicillin is illustrative [138]. Following a chance observation by Fleming [139] of the antibacterial activity of a contaminant mould spore, it was more than 10 years before the chemists Chain and Florey (with others) developed an effective production process for the active principle, including the equally serendipitous discovery of the role of corn-steep liquor in improving its yield. However, as pointed out before [138,140], the concept of antibiosis of bacteria by penicillia was not at all novel, having been observed 58 years previously by both Burdon Sanderson [141] and Lister [142], whereas a book by Papacostas & Gaté [143] had just been published containing a 60-page section, headed ‘Antibiosis’, on the extensive subject of bacterial inhibition by moulds and by other bacteria, devoted to previously published observations on this phenomenon and with several hundred historical references! From the perspective of an evolutionary metaphor, we recognize that part of the serendipitous process involves an actual recognition of a different fitness function from that originally used to construct the landscape (‘chance favours the prepared mind’ [144]), and how one might progress across the evolutionary landscape accordingly (i.e. by changing not only the *XY*-coordinates but also the fitness functions), as per ‘innovation by analogy;’ as discussed above under TRIZ.

Certainly, at least two significant scientific discoveries or innovations made by one of us (D.B.K.) came from experiments done with entirely different goals in mind. The first—a radio-frequency dielectric biomass probe [145,146], leading to the founding of a successful company (Aber Instruments, www.aber-instruments.co.uk)—came about through a search for experiments that might help one detect lateral ion transport along the surfaces of biological membranes, as part of a programme to test competing theories of membrane bioenergetics [147–151]. The second, the discovery of a bacterial cytokine or ‘wake-up molecule’ [152–156], came from studies on what happens to non-sporulating bacteria when we starve them [157–163], and certainly with no expectation that they might enter true dormancy [164] or secrete ‘wake-up’ signals such as the ‘Rpf’ factor that we found [152]; however, given its strongly antigenic nature [165], it is now under test as a vaccine candidate against mycobacteria.

Another example of a serendipitous discovery by the second author was the synthesis of novel hydroxyl-benzyl amino acid derivatives when by mistake instead of an omega-amino acid that was believed to be the right molecule to study membrane interactions, its alpha-derivative was used. This new compound proved to be a potential useful diagnostic tool to study secretory kidney function [166,167].

In addition to serendipity in scientific discoveries, some of the world's most iconic products happened by accident—such stories include for example pharmaceuticals and the food sector. Proscar was designed to treat the benign enlargement of the prostate. After 5 years on the market in the 1990s, it became clear that one of the side effects of Proscar was hair growth on bald men and this was immediately recognized as an opportunity to launch a new product, Propecia, to treat male-pattern baldness. Pfizer developed sildenafil as a treatment for hypertension, angina and other symptoms of heart disease. However, phase I clinical trials revealed that while the drug was not great at treating what it was supposed to treat, male test subjects were experiencing a rather unexpected side effect: erections. A few years later, in 1998, it was marketed as Viagra in the USA as a treatment for ‘erectile dysfunction’ and became an overnight success. It now turns over an estimated $1.9 billion dollars a year. The recognition of the frequency of drug promiscuity [168] is now a major and purposive activity, referred to as ‘drug repurposing’ [169–171].

Brandy started off as a by-product of transporting wine. About 900 years ago, merchants would essentially boil the water away from large quantities of wine in order both to transport it more easily and to save on customs taxes, which were levied by volume. After a while, a few of these merchants, bored perhaps after a long day on the road, dipped into their inventory and discovered that the concentrated, or distilled, wine actually tasted rather good and that is how brandy was born. Another food beverage, this time non-alcoholic, was originally invented as an alternative to morphine addiction, and to treat headaches and relieve anxiety. Coke's inventor, John Pemberton—a Confederate veteran of the Civil War who himself suffered from a morphine addiction—first invented a sweet, alcoholic drink infused with coca leaves for an extra kick. He called it Pemberton's French Wine Coca. It would be another two decades before that recipe was honed, sweetened, carbonated and eventually, marketed into what it is today Coca Cola.

## 7. So how can we foster innovation? ‘Directed evolution’ in innovation

### 7.1. What would it take to attain the virtue of innovation?

Serendipity is welcome, but is probably not the best strategy to rely upon. The question then becomes what type of innovation virtue (Platonic ‘ideal’) do we need to nurture—virtue or true virtue, incremental or disruptive innovation? There could be different schools of thoughts, with a tendency to concentrate only on disruptive innovation. However, we believe that it would be beneficial to have and foster both, and to be explicit about their pros and cons. The pros of incremental innovation are that it can generate news relatively quickly, be it via a scientific publication or the launch of a new product version. In addition to this, in dialectic terms, quantity can lead to quantity and when a critical number of incremental innovations are achieved, it can result in a major breakthrough. The cons of incremental innovation include that it can have a limited impact and may not last for very long (has limited memory) and will quickly be superseded by new developments. Disruptive innovation can though have a major impact and become a spring board for new developments, but because disruptive innovations are (almost by definition) rarer, disruptive innovation does require more time and ‘skill’ to develop. There are a number of ‘directive’ elements that can be applied for both incremental and disruptive innovation.

#### 7.1.1. Constructing the landscape

If we accept the metaphor of all kinds of ‘innovation’ as being equivalent, at least in part, to search on an ‘adaptive’ or ‘evolutionary’ landscape for improved fitness, it becomes comparatively easy to map the elements of what we need to do to improve such searches (and there is of course a considerable literature on the role of ‘genetic’ searches in all kinds of single and multi-objective optimization [14,44–46,172–188]). We thus need to consider the elements of evolutionary systems (as above) from the perspective of their mapping onto real-world phenomena.

#### 7.1.2. Selection and fitness

Equally important to the evolutionary process, and not entirely separable from the question of the maintenance of diversity in that it can contribute to it, is the nature of the selection regime. Peer review of grant applications and publications (leading to success in funding or publication) is an obvious example of selection in the academic world (and ‘alternative metrics' implying different selection or fitness regimes are widely discussed), whereas product successes in the market place are a metric of success for product innovation. It is also reasonable to consider the generation of novelty as ‘science push’ and the selection of that novelty as ‘market pull’.

While the fundamental elements are the same in achieving both types of virtues (incremental and disruptive innovations), the difference comes from the ‘landscape scope’ (diversity of the landscape) and the ‘how’ (how to navigate through the landscape).

#### 7.1.3. Landscape scope

Any breakthroughs require ‘out-of-the-box’ thinking that can be manifested in different ways. This even includes the construction or understanding of the landscape, for example an ability to construct a diverse landscape with the right fitness.

The maintenance of diversity to prevent premature convergence to suboptimal solutions is a well-known means of avoiding becoming trapped in local optima, and relates to the exploration/exploitation trade-off discussed above. This diversity is in both fitness (which is normally always measured) and in ‘genotype’ (which frequently is not [129]).

If much of the search involves changing or swapping the various components necessary for a good result, it is first and obviously necessary to *have* many of them, as well as means of retaining the best ones and creating new ones [14]. So *one recommendation is that to innovate one must read and experiment widely and try many combinations to achieve success* [189]. (This route, in fact, is part-and-parcel of Plato's suggestion of manipulating existing knowledge to various ends.) This implies a need for multi- and inter-disciplinarity, whether within individuals or in teams. There is also an implication that one should try multiple parallel experiments before selecting too early that which might prove most productive. There is evidence that this was the strategy used by Microsoft in the early development of what became the Windows operating systems for personal computers [190].

#### 7.1.4. Navigation through the landscape

Incremental innovation is typically characterized by local search, using exploitation to search the landscape, whereas disruptive innovation involves exploration (looking further afield, possibly with a greater risk of failure but also perhaps with a greater chance of a notable success). How best to navigate the landscape is in some sense a function of its ‘ruggedness’. ‘Ruggedness’ is a term that is hard to define exactly, but if there is a good correlation between fitness and ‘distance’ a landscape is said to be (relatively) smooth, whereas if fitness and distance are weakly correlated—with lots of mountains and valleys appearing seriatim as distance varies—then the landscape is more rugged. From this perspective, exploitation makes more sense for smooth landscapes, whereas exploration makes more sense for rugged landscapes.

The ability to broaden the landscape and a high degree of exploration creates opportunities to challenge the existing dogma and thus to lay the path to new discoveries. ‘History is replete with tragic and comic confrontations between experts fanatically attempting to enclose knowledge within a definitive representation of the world and discoverers stubbornly attempting to open, enlarge and disrupt it. This confrontational need to oppose dogmas and consecrated knowledge in order to achieve momentous discoveries did not always originate from improved hypotheses about the natural world. Certain major findings were not only made in spite of a misconception, sometimes they happened precisely because of it’ [191].

## 8. Concluding remarks

This is a broad subject, and in a short article, we cannot possibly hope to be comprehensive. However, we find that the evolutionary metaphor is indeed extremely (and possibly surprisingly) useful for understanding diverse processes of innovation both in science and industry. It is important to recognize from the beginning which path one wants to pursue—disruptive or incremental innovation. If one would decide to engage in the development of a disruptive innovation, be it in industry or science, two key aspects should be taken into consideration: time and landscape. As discussed in the article, disruptive innovation will have a bigger impact but it could take a long time, for example the recognition of most breakthrough innovations came many years later [192]. In terms of the landscape, incremental innovation follows a more gradual and specific path within given boundaries, while disruptive innovation, possibly in a way similar to so-called blue sky research starts ‘along a path but being free to branch out’ [192] and explore beyond the local landscape. Such a recognition implies that one can seek to promote creativity and innovation by adopting and adapting such evolutionary principles. While many centuries separate Plato and Darwin, it seems that Platonic virtue or true virtue may be evolved as well as attained.
